# A Reflection

**Published:** 1892-01-30

**Authors:** 


### PASSING TOPICS.

### A REFLECTION.

THE violent death of Tom, the Downing Street cat, friend and pet of a long line of statesmen, some of whom predeceased the redoubtable animal, while others survive to lament his untimely end, 's one of those events which arouse the imagination, not less than the sentiment, of mankind. Cats are not altogether unfamiliar things. To the inhabitants of certain cat-ridden neighbourhoods they have ceased to be objects of solicitude. By night and day they are unpleasantly prodigal of their presence, and they appeal not only to the eye, but to the ear in long-drawn notes of no particular sweetness.

We believe it has been a custom from the early days of the Blackwall Railway for the habitual travellers of that elevated line to cheer the monotony of their somewhat sombre journey by wagering as to the number of cats which can be discovered through the murky air roving on the roofs, while the desolation of many a suburban garden is directly attributable to feline depredations. But there are cats and cats, and these are of the common herd. We have no specific information as to the lineage of Tom of Downing Street: whether he came of a lordly stock and took naturally and as by right of birth to lofty courses, or whether, as is meet in a democratic age, he rose, by force of character, from a lowly origin to higher things, we do not know. But that he affected the society of Prime Ministers and Secretaries of State, as one to the manner born, there is evidence to show, and that he bore his dignities well many are ready to testify. Like breeds like, and the favourite of statesmen must of necessity incline to statesmen-like ways. For such an animal to know Downing Street was in itself a liberal education in state-craft, and although his friendships seemed impartially formed, one can imagine his diplomatic soul occasionally vexed with the vicissitudes of party strife, and that unavoidable interruption to repose which would naturally accompany a change of Government.

Whether it was possible that Lord Beaconsfield and Mr. Gladstone, who, we are told, have been among his patrons, rubbed him precisely in the same way we cannot tell, but it was good policy, and strictly constitutional to boot, to smile on all alike, and if at heart he favoured any party in particular, he was careful that his concern was openly accorded to nothing less than the Empire as a whole. It would have been less painful had the affection with which this great creature was regarded by mankind extended to their dogs, though in that case the epic of his life would have been incomplete, and we can well believe that had he been free to choose the manner of his death his martial spirit, so touchingly displayed in his choice of a sentry box as his home, would have gloried in the prospect that he would fall fighting valiantly against fearful odds. However this may be, the wanton and murderous act of the two bull terriers who, entirely insensible to his great qualities, did him to death after a prolonged conflict, have made his end glorious and furnished us with the reflection which we have had in mind all through this page. No matter how great or beneficent an institution may be, there are always some to assail it, and it is not for want of will but only for lack of power in tooth and claw that they fail to maim or kill. What a happy thing it is that malevolence cannot always command the weapons it aspires to. Spiteful people are never half thankful enough for their failures.

